# Analysis of Factors Affecting Quality of Life in Patients Treated for Maxillofacial Fractures

**DOI:** 10.3390/ijerph17010004

**Published:** 2019-12-18

**Authors:** Maciej Sikora, Mikołaj Chlubek, Elżbieta Grochans, Anna Jurczak, Krzysztof Safranow, Dariusz Chlubek

**Affiliations:** 1 Department of Maxillofacial Surgery, Hospital of the Ministry of the Interior, 25-375 Kielce, Poland; sikora-maciej@wp.pl; 2 Department of Biochemistry and Medical Chemistry, Pomeranian Medical University in Szczecin, 70-111 Szczecin, Poland; mikolaj.chlubek@gmail.com (M.C.); chrissaf@mp.pl (K.S.); dchlubek@pum.edu.pl (D.C.); 3 Department of Nursing, Pomeranian Medical University in Szczecin, 71-210 Szczecin, Poland; 4 Department of Clinical Nursing, Pomeranian Medical University in Szczecin, 71-210 Szczecin, Poland; anna.jurczak@pum.edu.pl

**Keywords:** maxillofacial fractures, quality of life, conservative treatment, hospitalization

## Abstract

Maxillofacial fractures (MFF) belong to the major modern medicine and public health concerns. The recovery from MFF is associated with a number of social problems. The patient’s mood may be affected by the change in self-image and lack of satisfaction with life, in many cases leading to a deepening of mental health disorders, resulting in alcoholism, loss of job or conflicts in the area of family life. The aim of this study was to evaluate the quality of life of patients with MFF, with respect to demographic and medical variables. The mean age of the 227 patients was 36 years. The mandible was the most frequent MFF location (52.9%), followed by the zygomatic bone (30.8%) then the maxilla (16.3%). Bone fracture displacement occurred in 79.3% of patients. A comminuted fracture was found in 71% of patients. The quality of life of patients with MFF was significantly better in all analyzed domains 3 months after the end of hospitalization compared to the initial survey carried out shortly after implementation of the treatment. Among the demographic variables, older age had a statistically significant but weak positive association with the improvement of the quality of life of respondents in General health perception domain.

## 1. Introduction

Maxillofacial fractures (MFF) are one of the major concerns of modern medicine and public health services. Their causes and frequency vary from region to region [1,2,3,4], and result from the socio-economic status of patients, their education, and cultural and environmental factors, as well as alcohol consumption [5,6,7]. According to the World Health Organization, maxillofacial injuries are most frequently caused by road collisions, violence, or sports injuries [2,4,7]. These may result in injuries to the soft tissues of the face and mouth, the teeth, and fractures of the craniofacial bones [1,3,4].

A significant source of trauma to the maxillofacial area is violence, defined as the deliberate use of physical force by an attacker against a victim [8]. Lee [9] showed that interpersonal violence (IPV) was the cause behind 44% of MFF cases in an 11-year observation period. Reports from Turkey, the Netherlands, North America, Bulgaria, Switzerland and South Africa also confirm that one of the most frequent causes of MFF is such physical violence [10,11,12,13,14,15]. Results of other studies cite alcohol abuse and the increased prevalence of aggression as dominant causes of MFF [16,17].

The best possible therapeutic outcome for MFF cases requires detailed diagnosis, planning and ensuring the timely restoration of the proper function and aesthetics of the traumatized organs, as well as appropriate physical, psychological and social rehabilitation. The occurrence of localized pain [18] and the treatment process, from surgical intervention [19] to rehabilitation [20], can have a significant impact on the quality of life of the patients. Apart from the physical consequences, current literature also emphasizes the importance of the psychological consequences of MFF [19,21,22], including post-traumatic stress disorder, depression and anxiety [23].

The recovery from MFF is associated with a number of social problems that need to be circumvented by working together in a therapeutic team. The patient’s mood may be affected by the change in self-image and lack of satisfaction with life, in many cases leading to a deepening of mental health disorders, resulting in alcoholism, loss of job or conflicts in the area of family life [24]. For this reason, it seems appropriate to study the quality of life of patients following MFF [19,25,26] so that the data obtained in this way may be helpful in the future in the implementation of programs supplementing the treatment process.

### Aim of the Study

With this in mind, we undertook a study to evaluate the quality of life of patients with MFF, with respect to demographics (gender, age) and medical variables (type and location of fracture).

## 2. Materials and Methods

The study was carried out in a group of 227 patients with maxillofacial injuries who were hospitalized at the Department of Maxillofacial Surgery of the Independent Public Health Care Facility of the Ministry of Interior and Administration in Kielce, Poland. Ambulatory care was provided at the Maxillofacial Surgery Outpatient Clinic “Ars Medica” in Kielce, Poland. The research project was supported by the Bioethics Committee of the Pomeranian Medical University in Szczecin and was carried out in accordance with the principles resulting from the Helsinki Declaration.

After admission to the ward, patients were subject to physical examination and interview. The necessary laboratory and radiological tests were carried out, as well as consultations with physicians of other specialties. After obtaining the necessary data, the patients were qualified for suitable trauma treatment. Depending on the extent of the injuries, degree of bone fracture displacement, existing functional and/or aesthetic disorders, general condition of the patient, and the patient’s decision, one of three basic therapeutic strategies was applied:

- conservative treatment,
- closed treatment
- open treatment.

The inclusive criteria for participation of the patient in the study were as follows:

- confirmation of medical diagnosis of a fracture of only one craniofacial bone,
- the moment of fracture not sooner than 10 days before admission to the hospital,
- the patient’s consent to participate in the study,
- maintenance of good verbal-logical contact with the patient.

The basis for excluding a patient from the study was any failure to meet any of the criteria for inclusion.

The quality of life of patients qualified for the study was assessed by means of a diagnostic survey using the standardized Quality of Life Assessment Questionnaire SF-36 v2, along with a questionnaire about sociodemographic data and selected treatment data.

The Quality of Life Questionnaire SF-36 consisted of 34 questions and enabled assessment of the quality of life in 9 domains: Physical functioning—PF, Role limitations due to physical problems—RP, Bodily pain—BP, General health perception—GH, Vitality—VT, Social functioning—SF, Mental health—MH, Role limitations due to emotional problems—RE and Self-Evaluated Transition—SET. The quality of life in each domain was expressed in a number ranging from 0 to 100. For the SF-36 there are no standards, so it is not possible to say whether the results achieved by the respondents would mean an objectively high or low quality of life. As such, values can be compared in individual domains/categories between distinguished groups of people, or to analyze changes over time in the same people and identify areas with the greatest differences or changes.

The quality of life of the patients with MFF was evaluated in two stages. The first survey was performed after the implementation of treatment, i.e., after the surgical procedure in open and closed treatment, and following the conservative treatment in those respective patients. The average length of hospitalization was 5.1 days (ranging from 2 to 12 days). The second examination was performed about 3 months after the patient was discharged from the hospital, during a follow-up visit to the maxillofacial surgery clinic.

### Statistical Analysis

Our study with 227 patients had 80% statistical power to detect differences between two measurements of quality of life parameters equal to ±5 points, assuming typical standard deviation of 25 for a difference between scores on 0–100 scale for each QoL domain. Program PS version 3.1.2 (http://biostat.mc.vanderbilt.edu/wiki/Main/PowerSampleSize) was used for the power calculation. The distribution of measurable variables significantly differed from a normal distribution (*p* < 0.05, Shapiro-Wilk test), therefore in the analysis, nonparametric tests were carried out: Mann-Whitney U test for comparisons between independent groups, and Wilcoxon signed rank test for comparisons of paired results of the two tests. To assess the relationship between measurable variables, a Spearman rank correlation coefficient was calculated. Arithmetic difference was calculated as an indicator of changes in the quality of life parameters immediately after the implementation of treatment and 3 months later. The average ± standard deviation and the median (interquartile range) are presented as descriptive statistics of the groups. Multifactor analysis was performed using a general linear model (GLM), presenting the values of regression coefficients for individual independent variables in the model and their 95% confidence intervals. The threshold of statistical significance was *p* < 0.05. The calculations were performed using Statistica v13 software (Dell Inc., Round Rock, TX, USA).

## 3. Results

The mean age of the 227 patients was 36 years. Women constituted 13.7% and men 86.3% of respondents. More than half of the patients lived in the countryside (55%), 24.7% in a big city and 21.3% in a small city.

**Figure 1:**
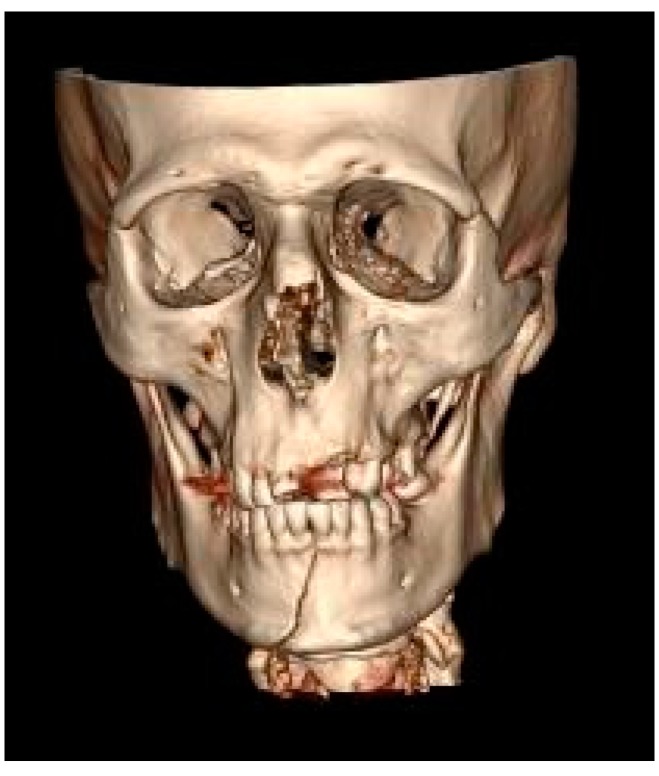
Mandibular symphyseal fracture (computed tomography with 3D reconstruction).

**Figure 2:**
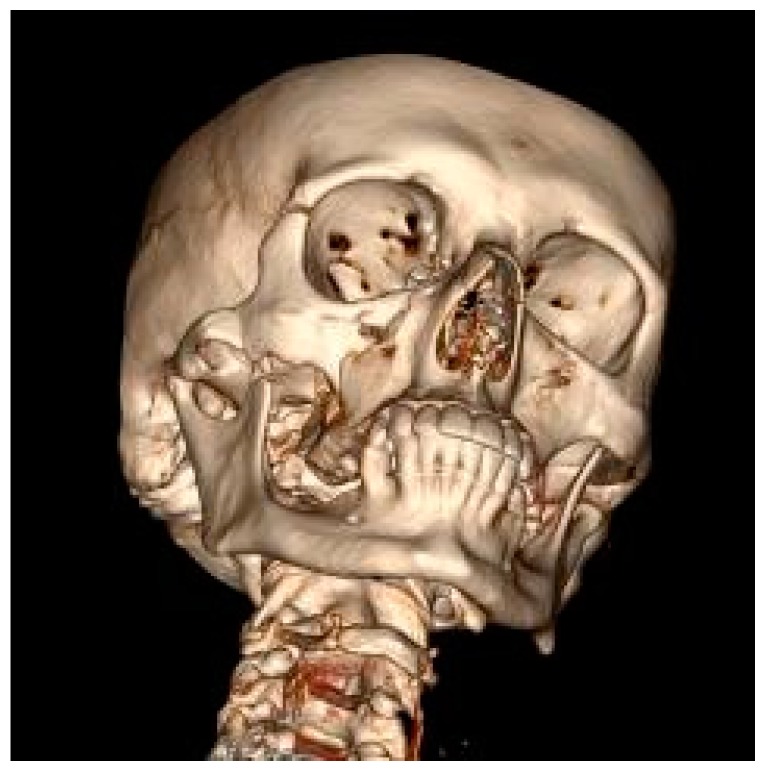
Right zygomatic bone fracture (computed tomography with 3D reconstruction).

**Figure 3:**
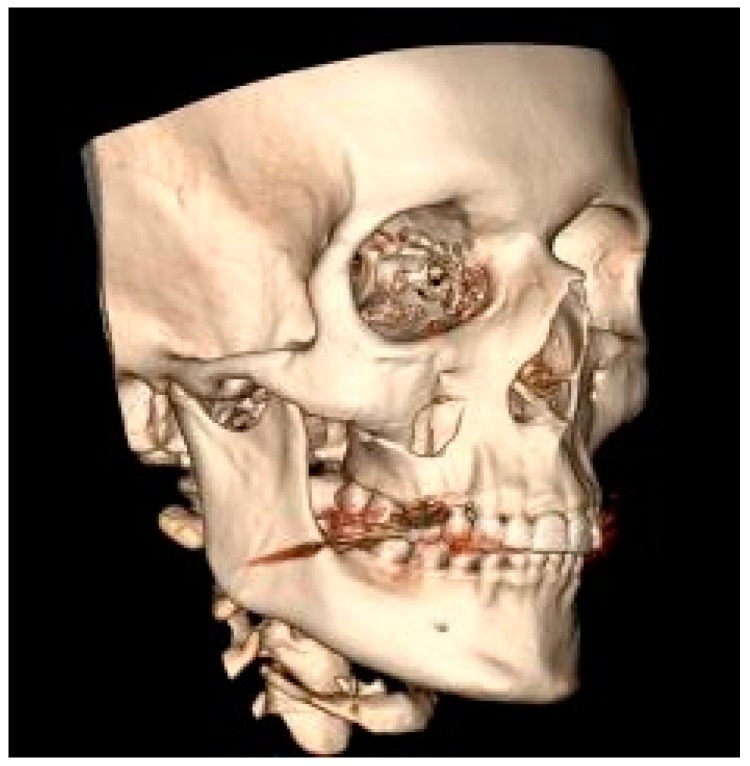
Fracture of the right maxilla (computed tomography with 3D reconstruction).

The mandible was the most frequent MFF location (52.9%) (Figure 1), followed by the zygomatic bone (30.8%) (Figure 2) then the maxilla (16.3%) (Figure 3).

Bone fracture displacement occurred in 79.3% of patients. A comminuted fracture was found in 71% of patients.

Most patients (78%) then received open treatment, 16.7% received closed treatment and 5.3% received conservative treatment.

Violence was the cause of the fracture in 61.7% of respondents. The remaining 38.3% were caused by traffic accidents, injuries at work or home, injuries suffered during epileptic seizures, or while practising sports.

Statistical analysis of the obtained data showed highly significant (*p* < 0.00001) differences in all domains of quality of life between the studied time points. The patients’ assessment in each domain was higher in the study conducted 3 months after the end of hospitalization than in the baseline study, and this improvement was measured by positive arithmetic differences in parameter values calculated individually for each patient, expressing an improvement in quality of life between the measurement conducted after the implementation of treatment and during the control visit 3 months later (Table 1).

A comparative analysis of the quality of life between patient genders was also carried out. On the basis of the QoL analysis, the first assessment, conducted after the implementation of treatment, revealed that men significantly better assessed their quality of life in the Physical functioning, Role physical, and Role emotional domains. A comparison of the QoL assessment 3 months later showed similar statistically significant differences in favor of men, but just in the Role physical and Role emotional domains. A comparison of the results of both studies showed no significant differences between the women and men, which implies that the improvement in quality of life during the observation period was not dependent on the gender of the patients (Table 2).

Another analysis concerned the relationship between QoL parameters and the age of the patients. In the case of QoL analysis after the implementation of treatment (first study), most domains (except Physical functioning and Vitality) correlated significantly negatively with age, which implies that the quality of life of the respondents generally deteriorated with age. The relationship between age and quality of life of patients 3 months after hospitalization was similar (second study). In this case, only the Vitality domain showed a statistically significant negative correlation. The difference between the results of both studies showed a significant positive correlation with age only in the General health perception domain, which implies a significantly greater improvement in GH in older patients (Table 3). It should be noted that while some mentioned above correlations of QoL parameters with age were statistically significant, they were generally weak (|Rs| ≤ 0.25), suggesting that the clinical importance of the associations is questionable.

The study also assessed the relationship between the quality of life of patients after MFF and clinical data describing the type of fracture (single or comminuted fracture). There were no statistically significant differences in any of the QoL domains in the first and second trials between the groups with single and comminuted fractures. However, there were significant differences between these groups concerning changes in the results between the first and second survey concerning Role limitations due to physical problems and Self-Evaluated Transition, which shows a significantly higher improvement in the quality of life in these domains in patients with comminuted fractures (Table 4).

Analyzing the relationship between QoL and variables describing the fracture site, i.e., distinguishing the fracture of the mandible and other localizations (maxilla or zygomatic bone), statistically significant differences were found in the fact that patients with a fracture of the mandible, assessed the quality of life after the implementation of treatment better than patients with a fracture with a different localization in the domains: Role limitations due to physical problems, Bodily pain and Social functioning. In the second study (3 months after the end of hospitalization) these differences were not confirmed. Analyzing the differences in QoL between the studies, significantly less improvement was found in the Role limitations due to physical problems and Social functioning in patients with a mandibular fracture (Table 5).

Multifactor analysis of the relationship between selected independent variables (gender, age, place of fracture) and individual quality of life domains of patients after MFF as dependent variables, showed that a maxilla fracture in comparison with other fractures is an age- and gender-independent factor associated with better quality of life in the Social functioning domain after the implementation of treatment (Table 6) and its smaller improvement after 3 months after the end of hospitalization (Table 7), and associated with better quality of life in the Bodily pain domain after the implementation of treatment (Table 8), but not with its improvement after 3 months after the end of hospitalization (Table 9). Age and gender in these models were not independent of the place of fracture and significant factors related to the quality of life after the implementation of treatment and its improvement 3 months after the end of hospitalization.

## 4. Discussion

The results of studies conducted so far confirm that men are more likely to suffer maxillofacial injuries than women [27,28], mostly in a fracture of the mandible [29], less frequently the fracture of the zygomatic bone, maxilla and orbits [30]. The etiology of craniofacial fractures varies. Some authors believe that the most common cause of these fractures are transport accidents [31], while others mention violence [3,30,32]. The type and severity of the injury largely depends on the studied population [33]. Young men are the most numerous risk group, often undertaking risky behaviour [3,34,35]. MFF is also relatively frequent among seniors, where it is mainly caused by falls [36]. Importantly, facial injuries may lead to stigmatization and a burden associated with everyday functioning in the physical and mental sphere, which in turn significantly affects the quality of everyday life [37].

A study carried out by Tamme et al. [38], which aimed to gather information on quality of life following orthodontic and surgical treatment, showed a deterioration in Physical functioning, General health and Mental health in comparison with the control group. There were no differences in the Bodily pain, Vitality, Social functioning, Role physical and Role emotional domains. However, in the research presented in this paper, statistically significant differences were observed in all the analyzed areas of quality of life. These differences consisted of improvements three months after the end of hospitalization compared to the initial study carried out shortly after the implementation of treatment. This result indicates the necessity to undertake the fastest possible actions to restore the efficiency in all areas of a patient’s functioning, i.e., functional, aesthetic and mental. Role limitation resulting from emotional problems (the Role emotional domain) is an important factor influencing the scope of functioning in the family and social space. A study by Zingler et al. [39], aimed at evaluating biological and psychological changes associated with orthognathic surgery in a surgery-first model, showed, among other things, a significant improvement in the quality of life in the third month after surgery compared to the initial phase of treatment. The parameters that improved were the social aspect, aesthetics and functioning of the oral cavity.

The study conducted by Omeje et al. in a group of 56 people with a broken mandible showed that patients qualified for ORIF (Open Reduction and Internal Fixation) treatment reported higher values in the Bodily Pain domain, whereas patients treated with Maxillo-Mandibular Fixation indicated higher values in the physical and psychosocial domains [40]. Another study by Omeje et al. was an analysis of quality of life after treatment of fractures in the mandible. The study used the Geriatric/General Oral Health Assessment Index, which consists of three domains: Physical (eating, speaking, swallowing), Psychosocial (oral health concerns, dissatisfaction with appearance, self-awareness of oral health, and social contacts) and Pain (use of medication and discomfort). The results reflected an improvement in the quality of life indicators in relation to the time elapsed since the surgical procedure, except for the first day after the operation, when the quality of life indicators did deteriorate. The treatment method had no significant influence on the results of the study [41].

Similar results were obtained by Kaukola et al. in a study conducted in 79 patients with zygomatic bone fracture, using the 15-D assessment tool, which covers physical, mental and social aspects of health [26]. They indicate a deterioration of quality of life indicators on the first day after surgery and concern 6 out of 15 domains, including Vitality and Everyday Activity. In a retrospective study conducted in 45 patients with a broken mandible, Kaukola et al. demonstrated that the quality of life resulting from the state of health, at first significantly decreased and then improved within a few months after surgery [33]. In our study the quality of life in terms of Physical functioning and limitations in usual role activities because of physical health and emotional problems (Role physical and Role emotional) after the implementation of treatment (first study) was initially better in the men than in women. In contrast, three months after the end of hospitalization, the gender related differences were limited to the Role physical and Role emotional domains, and gender was not a significant factor for improving the quality of life between the two surveys.

Confronte et al. carried out an evaluation of the impact of surgical treatment in three time measurements on the quality of life of patients with MFF and oral trauma [19]. The study used Oral Health Impact Profile (OHIP-14). Injuries suffered by patients had the greatest impact on the quality of life immediately after the diagnosis was made. In addition, it was found that surgical treatment most effectively improved quality of life within 3 months of the implementation of therapy directed at multiple craniofacial and mandibular fractures.

Girotto et al. performed a cohort retrospective study on maxilla fractures according to the Le Fort classification [42]. The data collected over a period of eight years covered general health, somatic symptoms and psychosocial aspects. The studied group was characterized by indicators of general health condition similar to the group of patients with general injuries. There was a direct relationship between the severity of patients’ facial injuries and reported work disability. Only 55 and 58 percent of Le Fort patients from groups C and D (severely comminuted fractures), respectively had returned to work at the time of follow-up interview. These figures are significantly lower than the back-to-work percentage of patients with less severe facial injuries (70 percent).

A study of quality of life following orbital and other facial injuries conducted by Sharma and Kaur revealed a deterioration of patient physical, social and mental health [43]. Type II and III maxilla fractures according to Le Fort caused visual impairment and resulted in a deterioration in overall health. There was also a significant reduction in daily activities. Our own study evaluated quality of life with regard to the type or location of fracture after the implementation of treatment and 3 months after the end of hospitalization, and did not show any significant differences in the majority of domains analyzed. However, like other authors, significantly lower results were found in self-assessment and the Role physical domain, which proves that the type of fracture has a significant impact on the quality of life in terms of social and psychological status.

MFFs are complex medical problems, often leading to deformities, impairment of function in the injury area, development of chronic disease, as well as psychological consequences such as anxiety and depression [44]. Fractures in the central part of the face also increase the risk of chronic sinusitis. The accompanying symptoms cause long-term negative effects, which significantly affect the quality of life of patients [45]. The results of a survey conducted by Gironda et al. in a group of people suffering from fractures of maxillary bones revealed that depressive symptoms intensified in the early postoperative period as a result of pain [46]. A literature review by Sahni [21] indicates a link between facial injury and more frequent occurrence of mental disorders (including generalized anxiety disorders and post-traumatic stress syndrome), especially in attack victims, which significantly deteriorates their quality of life. In addition, the results of a study conducted by Hull et al. emphasizes the adverse effect of MFF on mental functions of patients, both immediately after the event and 4–6 weeks later [47]. In our own research on the relationship between the location of the fracture and quality of life, we found the reported quality of life was higher in patients with a mandibular fracture, in the Role physical, as well as Bodily pain and Social functioning domains. However, these differences were not confirmed in a survey conducted 3 months later. In addition, we found significantly lower improvement in the Role physical and the Social functioning domains in patients with a mandibular fracture.

## 5. Conclusions

The quality of life of patients with MFF was significantly better in all analyzed domains 3 months after the end of hospitalization compared to the initial survey carried out shortly after implementation of the treatment. Among the demographic variables, age and gender had a significant impact on the improvement of the quality of life of respondents in specific domains. Men rated their quality of life higher (in the areas of the Role physical and the Role emotional domains), and younger people in all domains except Vitality. Comminuted fractures were associated with a greater improvement in the quality of life in the Role physical domain and self-evaluated transition in comparison to single fractures. Patients with a fractured mandible rated their quality of life higher after treatment than patients with other types of fractures, in the domains of Bodily pain, Role physical, and Social functioning. This concerned the difference between the surveys, and was indirectly due to the higher levels of reported QoL in the first survey reported by patients with mandibular fractures.

## Figures and Tables

**Table 1:** Quality of life domains after the implementation of treatment (first study) and 3 months after termination of hospitalization (second study) and significance of differences between the results of both surveys.

QoL Domain	First Survey		Second Survey		Difference between the Results of the Surveys		Statistical Significance of the Difference *p* *
	M ± SD	Me (IQR)	M ± SD	Me (IQR)	M ± SD	Me (IQR)	
Physical functioning	73.1 ± 25.7	80 (35)	92.5 ± 16.6	100 (5)	19.1 ± 25.1	15 (30)	<0.00001
Role limitations due to physical problems	56.3 ± 29.7	56.3 (43.8)	79.8 ± 21	81.3 (31.3)	23.3 ± 28.4	25 (43.8)	<0.00001
Bodily pain	44.7 ± 26.6	44.4 (33.3)	81.4 ± 23.9	88.9 (22.2)	36.7 ± 29.1	44.4 (44.4)	<0.00001
General health perception	55.8 ± 21	55 (25)	61.1 ± 18	60 (25)	5.3 ± 13.5	5 (10)	<0.00001
Vitality	51 ± 21	50 (37.5)	62.3 ± 16.9	62.5 (25)	11.2 ± 20.1	6.3 (25)	<0.00001
Social functioning	53.9 ± 30.8	50 (50)	82.9 ± 22	87.5 (25)	29 ± 30.6	25 (50)	<0.00001
Role limitation due to emotional problems	62.5 ± 30.6	66.7 (58.3)	82.7 ± 21.6	91.7 (25)	20.1 ± 29	16.7 (33.3)	<0.00001
Mental health	53.3 ± 20.8	55 (30)	67.2 ± 15.8	70 (20)	14.1 ± 19.7	10 (25)	<0.00001
Self-Evaluated Transition	34.6 ± 23.1	25 (25)	62 ± 21.1	50 (25)	27.3 ± 25.4	25 (50)	<0.00001

* Wilcoxon signed rank test. QoL—quality of life; M—mean; SD—standard deviation; Me—median; IQR—interquartile range.

**Table 2:** Comparison of the quality of life of patients after implementation of treatment (first study) and 3 months after termination of hospitalization (second study) between the women and men.

QoL Domain	Women (n = 31)		Men (n = 196)		Statistical Significance of the Difference *p* *
	M ± SD	Me (IQR)	M ± SD	Me (IQR)	
**First survey**					
Physical functioning	66.5 ± 23.6	75 (25)	74.2 ± 25.9	80 (30)	0.02
Role limitations due to physical problems	44.4 ± 26.1	50 (37.5)	58.2 ± 29.8	56.3 (50)	0.02
Bodily pain	38.7 ± 17.9	33.3 (22.2)	45.7 ± 27.6	44.4 (44.4)	0.27
General health perception	51.5 ± 18.9	50 (25)	56.5 ± 21.3	55 (27.5)	0.12
Vitality	46.4 ± 18.2	50 (18.8)	51.8 ± 21.3	50 (37.5)	0.18
Social functioning	45.2 ± 26.9	37.5 (37.5)	55.3 ± 31.2	62.5 (62.5)	0.08
Role limitation due to emotional problems	48.9 ± 29.4	50 (50)	64.7 ± 30.3	66.7 (50)	<0.01
Mental health	49 ± 19.5	50 (30)	54 ± 21	55 (30)	0.18
Self-Evaluated Transition	28.2 ± 18	25 (25)	35.6 ± 23.7	25 (25)	0.11
**Second survey**					
Physical functioning	87.4 ± 23.2	100 (10)	93.3 ± 15.3	100 (5)	0.09
Role limitations due to physical problems	74.2 ± 19.5	75 (37.5)	80.6 ± 21.2	81.3 (25)	0.04
Bodily pain	80.3 ± 21.8	88.9 (33.3)	81.6 ± 24.3	88.9 (22.2)	0.41
General health perception	56.3 ± 16.3	55 (15)	61.8 ± 18.2	60 (25)	0.06
Vitality	60.9 ± 14.8	62.5 (25)	62.5 ± 17.2	62.5 (25)	0.72
Social functioning	82.7 ± 21.1	87.5 (37.5)	82.9 ± 2.2	87.5 (25)	0.77
Role limitation due to emotional problems	75.8 ± 21.2	75 (50)	83.8 ± 21.5	95.8 (25)	0.02
Mental health	63.1 ± 15.3	65 (25)	67.8 ± 15.8	70 (20)	0.08
Self-Evaluated Transition	61.3 ± 18.1	50 (25)	62.1 ± 21.6	62.5 (25)	0.68
**Difference between the results of the two surveys**					
Physical functioning	21 ± 29.3	20 (25)	18.8 ± 24.4	15 (30)	0.40
Role limitations due to physical problems	29.8 ± 25.6	31.3 (50)	22.2 ± 28.7	25 (43.8)	0.15
Bodily pain	41.6 ± 28	44.4 (33.3)	35.9 ± 29.3	44.4 (44.4)	0.31
General health perception	5 ± 11.1	5 (10)	5.3 ± 13.8	5 (10)	0.65
Vitality	14.5 ± 21.3	12.5 (31.3)	10.6 ± 19.9	6.3 (25)	0.24
Social functioning	37.5 ± 32.3	37.5 (50)	27.6 ± 30.1	25 (50)	0.12
Role limitation due to emotional problems	26.9 ± 28.8	16.7 (50)	19 ± 28.9	16.7 (33.3)	0.30
Mental health	14 ± 19.8	10 (20)	14.1 ± 19.7	10 (25)	0.96
Self-Evaluated Transition	33.1 ± 24.5	25 (25)	26.3 ± 25.5	25 (50)	0.22

* Mann-Whitney U test. QoL—quality of life; M—mean; SD—standard deviation; Me—median; IQR—interquartile range.

**Table 3:** Correlation between the quality of life of patients in the study after treatment implementation (first study) and 3 months after termination of hospitalization (second study) and the difference between the results of both studies, with the age of the patients.

QoL Domain	Correlation with Age	
	Rs	*p* *
**First survey**		
Physical functioning	−0.12	0.06
Role limitations due to physical problems	−0.16	0.02
Bodily pain	−0.14	0.04
General health perception	−0.25	<0.01
Vitality	−0.10	0.12
Social functioning	−0.14	0.03
Role limitation due to emotional problems	−0.22	<0.01
Mental health	−0.19	<0.01
Self-Evaluated Transition	−0.16	0.02
**Second survey**		
Physical functioning	−0.13	0.04
Role limitations due to physical problems	−0.14	0.03
Bodily pain	−0.18	0.01
General health perception	−0.18	0.01
Vitality	−0.23	<0.01
Social functioning	−0.09	0.20
Role limitation due to emotional problems	−0.18	<0.01
Mental health	−0.21	<0.01
Self-Evaluated Transition	−0.01	0.89
**Difference between the results of the two surveys**		
Physical functioning	0.11	0.12
Role limitations due to physical problems	0.08	0.21
Bodily pain	0.03	0.63
General health perception	0.20	<0.01
Vitality	−0.04	0.56
Social functioning	0.11	0.10
Role limitation due to emotional problems	0.13	0.06
Mental health	0.04	0.53
Self-Evaluated Transition	0.13	0.06

* statistical significance for Spearman’s Rank Correlation Coefficient (Rs). QoL—quality of life.

**Table 4:** Comparison of patient quality of life after implementation of treatment (first study) and 3 months after termination of hospitalization (second study) between patients with single and comminuted fractures.

QoL Domain	Single Fracture (n = 66)		Comminuted Fracture (n = 161)		Statistical Significance of the Difference *p* *
	M ± SD	Me (IQR)	M ± SD	Me (IQR)	
**First survey**					
Physical functioning	72.2 ± 28.8	80 (35)	73.5 ± 24.3	80 (32.5)	0.80
Role limitations due to physical problems	60.7 ± 29.2	62.5 (50)	54.4 ± 29.8	50 (50)	0.14
Bodily pain	48.1 ± 27.7	44.4 (44.4)	43.2 ± 26.1	44.4 (33.3)	0.19
General health perception	55.9 ± 23	55 (32.5)	55.8 ± 20.2	55 (25)	0.95
Vitality	50.9 ± 22.2	50 (31.3)	51.1 ± 20.5	50 (31.3)	0.93
Social functioning	59.1 ± 30.2	62.5 (50)	51.7 ± 30.9	50 (50)	0.10
Role limitation due to emotional problems	67.9 ± 28.2	70.8 (50)	60.3 ± 31.4	62.5 (58.3)	0.13
Mental health	53.8 ± 19.9	55 (30)	53.1 ± 21.3	55 (30)	0.81
Self-Evaluated Transition	36.7 ± 24.1	50 (25)	33.7 ± 22.7	25 (25)	0.33
**Second survey**					
Physical functioning	89.5 ± 20.9	100 (5)	93.7 ± 14.5	100 (5)	0.73
Role limitations due to physical problems	77.1 ± 24.5	75 (31.3)	80.8 ± 19.5	81.3 (25)	0.44
Bodily pain	81.5 ± 25.1	88.9 (22.2)	81.4 ± 23.5	88.9 (22.2)	0.62
General health perception	60.8 ± 19	60 (25)	61.2 ± 17.7	60 (25)	0.94
Vitality	62.5 ± 17.6	62.5 (25)	62.2 ± 16.7	62.5 (25)	0.69
Social functioning	82.7 ± 24.3	87.5 (25)	83 ± 21.1	87.5 (25)	0.77
Role limitation due to emotional problems	82.3 ± 24.1	100 (25)	82.9 ± 20.6	91.7 (25)	0.66
Mental health	66.2 ± 17.4	70 (25)	67.6 ± 15.1	70 (20)	0.96
Self-Evaluated Transition	58.1 ± 22.1	50 (25)	63.6 ± 20.6	75 (25)	0.12
**Difference between the results of the two surveys**					
Physical functioning	16.6 ± 26.1	15 (30)	20.1 ± 24.7	15 (30)	0.37
Role limitations due to physical problems	16 ± 27.2	12.5 (31.3)	26.3 ± 28.4	25 (50)	0.04
Bodily pain	33 ± 27.4	33.3 (44.4)	38.2 ± 29.7	44.4 (38.9)	0.09
General health perception	4.8 ± 14.4	5 (10)	5.5 ± 13.1	0 (10)	0.56
Vitality	11.1 ± 20.6	6.3 (25)	11.2 ± 20	6.3 (25)	0.90
Social functioning	23.3 ± 25.5	25 (37.5)	31.4 ± 32.2	25 (62.5)	0.12
Role limitation due to emotional problems	14.1 ± 25.3	8.3 (25)	22.6 ± 30.1	25 (41.7)	0.07
Mental health	12.5 ± 19.1	10 (20)	14.8 ± 19.9	15 (30)	0.56
Self-Evaluated Transition	21.2 ± 20.8	25 (25)	29.8 ± 26.7	25 (50)	0.03

* Mann-Whitney U test. QoL—quality of life; M—mean; SD—standard deviation; Me—median; IQR—interquartile range.

**Table 5:** Comparison of quality of life of patients after implementation of treatment (first study) and 3 months after termination of hospitalization (second study) between patients with mandibular fractures and other fracture locations.

QoL Domain	Maxilla or Zygomatic Bone (n = 107)		The Mandible (n = 120)		Statistical Significance of the Difference *p* *
	M ± SD	Me (IQR)	M ± SD	Me (IQR)	
**First survey**					
Physical functioning	71.5 ± 25.7	80 (35)	74.7 ± 25.7	80 (30)	0.23
Role limitations due to physical problems	52.2 ± 27.7	50 (50)	60 ± 30.9	62.5 (50)	0.03
Bodily pain	39.9 ± 24.7	33.3 (27.8)	49.1 ± 27.7	44.4 (44.4)	0.02
General health perception	54.4 ± 20.5	50 (25)	57.1 ± 21.5	55 (30)	0.44
Vitality	49.1 ± 19.6	50 (31.3)	52.9 ± 22	50 (31.3)	0.14
Social functioning	47.1 ± 29.8	43.8 (43.8)	59.8 ± 30.7	62.5 (50)	< 0.01
Role limitation due to emotional problems	59.1 ± 29.8	58.3 (50)	65.9 ± 31.1	75 (50)	0.07
Mental health	51.1 ± 21.1	50 (35)	55.2 ± 20.5	60 (25)	0.09
Self-Evaluated Transition	33.1 ± 24.1	25 (25)	35.8 ± 22.3	25 (25)	0.33
**Second survey**					
Physical functioning	91.5 ± 17.7	100 (5)	93.3 ± 15.7	100 (5)	0.30
Role limitations due to physical problems	78.7 ± 20.7	75 (31.3)	80.8 ± 21.5	81.3 (25)	0.28
Bodily pain	79.1 ± 25.6	88.9 (33.3)	83.3 ± 22.4	88.9 (22.2)	0.21
General health perception	61.6 ± 17.8	60 (25)	60.7 ± 18.4	60 (25)	0.68
Vitality	61.3 ± 17	62.5 (25)	63.3 ± 16.8	62.5 (25)	0.28
Social functioning	82.2±22.4	87.5 (25)	83.5 ± 21.8	87.5 (25)	0.78
Role limitation due to emotional problems	80.3 ± 22.3	83.3 (33.3)	85 ± 21	100 (25)	0.10
Mental health	66.1 ± 14.8	70 (20)	68.3 ± 16.6	75 (20)	0.11
Self-Evaluated Transition	61.1 ± 24.2	50 (25)	62.7 ± 18.1	75 (25)	0.60
**Difference between the results of the two surveys**						
Physical functioning	20 ± 25.4	15 (25)	18.2 ± 25	15 (30)	0.39
Role limitations due to physical problems	26.4 ± 25	25 (31.3)	20.4 ± 30.8	18.8 (43.8)	0.05
Bodily pain	39.6 ± 26.7	44.4 (33.3)	33.9 ± 30.9	33.3 (44.4)	0.15
General health perception	7.2 ± 11.8	5 (15)	3.6 ± 14.7	0 (10)	0.08
Vitality	12.4 ± 18.5	12.5 (25)	10.1 ± 21.5	6.3 (31.3)	0.21
Social functioning	35.6 ± 29.1	37.5 (50)	23.2 ± 30.9	12.5 (43.8)	<0.01
Role limitation due to emotional problems	21.2 ± 28.8	25 (41.7)	18.9 ± 29.2	16.7 (33.3)	0.33
Mental health	15.3 ± 19.1	15 (30)	13.3 ± 20.1	10 (25)	0.38
Self-Evaluated Transition	27.9 ± 26.8	25 (50)	26.7 ± 24.3	25 (50)	0.87

* Mann-Whitney U test *. QoL—quality of life; M—mean; SD—standard deviation; Me—median; IQR—interquartile range.

**Table 6:** Multifactor analysis of Social functioning domain after implementation of treatment (first study) as a dependent variable.

	Coefficients in GLM *			
Independent Variables	Value	−95% CI	+95% CI	*p*
Male gender	8.49	−3.31	20.29	0.16
Age [years]	−0.19	−0.48	0.09	0.18
Fracture of the mandible	12.02	3.95	20.09	<0.01

GLM—general linear model (R^2^ = 0.06, *p* < 0.01 for the whole model); CI—confidence interval.

**Table 7:** Multifactor analysis of the Social functioning domain (difference in QoL between the first and second surveys) as a dependent variable.

	Coefficients in GLM *			
Independent Variables	Value	−95% CI	+95% CI	*p*
Male gender	−9.16	−20.95	2.63	0.13
Age [years]	0.10	−0.18	0.39	0.47
Fracture of the mandible	−12.08	−20.16	−4.01	<0.01

GLM—general linear model (R^2^ = 0.06, *p* < 0.01 for the whole model); CI—confidence interval.

**Table 8:** Multifactorial analysis of Bodily pain after implementation of treatment (first study) as a dependent variable.

	Coefficients in GLM *			
Independent Variables	Value	−95% CI	+95% CI	*p*
Male gender	4.97	−5.28	15.22	0.34
Age [years]	−0.23	−0.47	0.02	0.07
Fracture of the mandible	8.32	1.32	15.32	0.02

GLM—general linear model (R^2^ = 0.05, *p* = 0.01 for the whole model); CI—confidence interval.

**Table 9:** Multifactorial analysis of the Bodily pain domain (difference in QoL between the first and second study) as a dependent variable.

	Coefficients in GLM *			
Independent Variables	Value	−95% CI	+95% CI	*p*
Male gender	−5.24	−16.66	6.19	0.37
Age [years]	0.07	−0.20	0.35	0.61
Fracture of the mandible	−5.45	−13.27	2.36	0.17

GLM—general linear model (R^2^ = 0.02, *p* = 0.33 for the whole model); CI—confidence interval.
